# Deletion of all three MAP kinase genes results in severe defects in stress responses and pathogenesis in *Fusarium graminearum*

**DOI:** 10.1007/s44154-021-00025-y

**Published:** 2022-01-17

**Authors:** Jingyi Ren, Yuhan Zhang, Yuhua Wang, Chengliang Li, Zhuyun Bian, Xue Zhang, Huiquan Liu, Jin-Rong Xu, Cong Jiang

**Affiliations:** 1 State Key Laboratory of Crop Stress Biology for Arid Areas and NWAFU-Purdue Joint Research Center, College of Plant Protection, Northwest A&F University, Yangling, 712100 Shaanxi China; 2 Department of Botany and Plant Pathology, Purdue University, West Lafayette, IN 47907 USA

**Keywords:** Abiotic stresses, Fungal-bacterial interaction, *Gibberella zeae*, Mycoparasitism, Signal transduction

## Abstract

Mitogen-activated protein kinase (MAPK) cascades are activated by external stimuli and convert signals to cellular changes. Individual MAPKs have been characterized in a number of plant pathogenic fungi for their roles in pathogenesis and responses to biotic or abiotic stresses. However, mutants deleted of all the MAPK genes have not been reported in filamentous fungi. To determine the MAPK-less effects in a fungal pathogen, in this study we generated and characterized mutants deleted of all three MAPK genes in the wheat scab fungus *Fusarium graminearum*. The *Gpmk1 mgv1 Fghog1* triple mutants had severe growth defects and was non-pathogenic. It was defective in infection cushion formation and DON production. Conidiation was reduced in the triple mutant, which often produced elongated conidia with more septa than the wild-type conidia. The triple mutant was blocked in sexual reproduction due to the loss of female fertility. Lack of any MAPKs resulted in an increased sensitivity to various abiotic stress including cell wall, osmotic, oxidative stresses, and phytoalexins, which are likely related to the defects of the triple mutant in environmental adaptation and plant infection. The triple mutant also had increased sensitivity to the biocontrol bacterium *Bacillus velezensis* and fungus *Clonostachys rosea*. In co-incubation assays with *B. velezensis*, the *Gpmk1 mgv1 Fghog1* mutant had more severe growth limitation than the wild type and was defective in conidium germination and germ tube growth. In confrontation assays, the triple mutant was defective in defending against mycoparasitic activities of *C. rosea* and the latter could grow over the mutant but not wild-type *F. graminearum*. RNA-seq and metabolomics analyses showed that the MAPK triple mutant was altered in the expression of many ATP-binding cassette (ABC) and major facilitator superfamily (MFS) transporter genes and the accumulation of metabolites related to arachidonic acid, linoleic acid, and alpha-linolenic acid metabolisms. Overall, as the first study on mutants deleted of all three MAPKs in fungal pathogens, our results showed that although MAPKs are not essential for growth and asexual reproduction, the *Gpmk1 mgv1 Fghog1* triple mutant was blocked in plant infection and sexual reproductions. It also had severe defects in responses to various abiotic stresses and bacterial- or fungal-fungal interactions.

**Supplementary Information:**

The online version contains supplementary material available at 10.1007/s44154-021-00025-y.

## Introduction

The homothallic ascomycete *Fusarium graminearum* is a causal agent of Fusarium Head Blight (FHB), a destructive disease of wheat, barley, and other grain cereals worldwide (Harris et al., 2016). Under favorable environmental conditions, infection of wheat or barley heads by *F. graminearum* often results in severe yield losses, grain quality reduction, and contamination of mycotoxins such as deoxynivalenol (DON) and zearalenone (Audenaert et al., 2013; Goswami and Kistler 2004). The fungus overwinters and forms perithecia on infected plant debris. Ascospores forcefully released from perithecia are the primary inoculum and *F. graminearum* infection can occur from anthesis to kernel filling. DON is an important virulence factor in this pathogen and *TRI* genes responsible for DON biosynthesis are expressed in infection cushions (Boenisch and Schafer 2011). For asexual reproduction, it produces macroconidia or conidia, which also are infectious and important for spreading infection and colonization of plant tissues. To survive in the nature and infect wheat or barley spikelets, *F. graminearum* must be able to sense various environmental and plant signals for properly regulating various developmental and infection production as well as DON production (Dilks et al., 2019; Jiang et al., 2019).

Like in other eukaryotic organisms, mitogen-activated protein (MAP) kinases play important roles in activating cellular responses to extracellular signals, including host and environmental stimuli in plant pathogenic fungi (Jiang et al., 2018a). In general, signals sensed by specific receptors are relayed to MAP kinase (MAPK) cascades to activate downstream transcription factors for regulating changes in gene expression (Gu et al., 2015; Jiang et al. 2019; Wang et al., 2015; Yun et al., 2014). Unlike the budding yeast *Saccharomyces cerevisiae* that has five MAPK genes involved in regulating pheromone response, filamentation, cell wall integrity, osmoregulation, and ascospore formation (Posas et al., 1998), *F. graminearum* and most ascomycetous phytopathogenic fungi have only three MAPKs, which are orthologous to yeast Fus3/Kss1, Slt2, and Hog1 (Li et al., 2012). The first MAPK gene characterized in *F. graminearum* is *MGV1*, an ortholog of yeast *SLT2* cell wall integrity MAPK and *MPS1* of the rice blast fungus *Magnaporthe oryzae* (Bermejo et al., 2008; Hou et al., 2002; Xu et al., 1998). The *mgv1* mutant was significantly reduced in growth rate and hypersensitive to cell wall stress. Mutants deleted of *MGV1* or its upstream MAPK kinase (MEK) and MEK kinase (MEKK) genes are non-pathogenic and sterile, and have severe cell wall defects (Hou et al. 2002; Wang et al., 2011; Yun et al. 2014). Deletion of *GPMK1* or *MAP 1*, an ortholog of *M. oryzae PMK1* also results in defects in DON production and sexual reproduction (Jenczmionka et al., 2003; Urban et al., 2003). Gpmk1 in *F. graminearum* also regulates the early induction of extracellular endoglucanase, xylanolytic, and proteolytic activities (Jenczmionka and Schafer 2005). Unlike the orthologs of *MGV1* and *GPMK1* that are essential for plant infection in general, the HOG pathway has a conserved role in osmoregulation but species-specific functions in pathogenesis in fungal pathogens (Jiang et al. 2018a; Zhang et al., 2021). Whereas its ortholog in *M. oryzae* is dispensable for virulence, *FgHOG1* is essential for plant infection in *F. graminearum.* Besides regulating responses to hyperosmotic and oxidative stresses and resistance against fludioxonil, the FgHog1 MAPK pathway is also involved in the regulation of DON biosynthesis and sexual reproduction (Wang et al. 2011; Zheng et al., 2012). Interestingly, null mutations in *FgHOG1* partially rescue the defect of the *mgv1* mutant in growth and cell wall integrity but not pathogenesis (Ren et al., 2019).

Although individual MAPK genes have been characterized for their regulatory roles in pathogenesis, development, and stress responses in a number of plant pathogenic fungi, to date, there is no report on mutants deleted of all three MAPK genes in any fungal pathogen. In *F. graminearum*, the Gpmk1, Mgv1, and FgHog1 MAPKs have distinct functions but they are all important for plant infection, DON production, and sexual reproduction, suggesting their overlapping roles in pathogenesis, development, and secondary metabolism. To further characterized the relationship among these three MAPK pathways, in this study we generated and characterized mutants deleted of *GPMK1, MGV1,* and *FgHOG1*. The *Gpmk1 mgv1 Fghog1* triple deletion mutant was viable but had severe growth defects. It was non-pathogenic and female sterile. Besides increased sensitivity to a variety of abiotic stresses, the triple mutant had increased sensitivity to biocontrol agents *Bacillus velezensis* and *Clonostachys rosea*. To our knowledge, this is the first study on mutants deleted of all three MAPKs in fungal pathogens, and our results showed that although MAPKs are not essential for growth and asexual reproduction, the *Gpmk1 mgv1 Fghog1* triple mutant was blocked in plant infection and sexual reproductions. It also had severe defects in responses to various abiotic stresses and bacterial- or fungal-fungal interactions.

## Results

### The *Gpmk1 mgv1 Fghog1* triple mutant is defective in hyphal growth and development

**Fig. 1:**
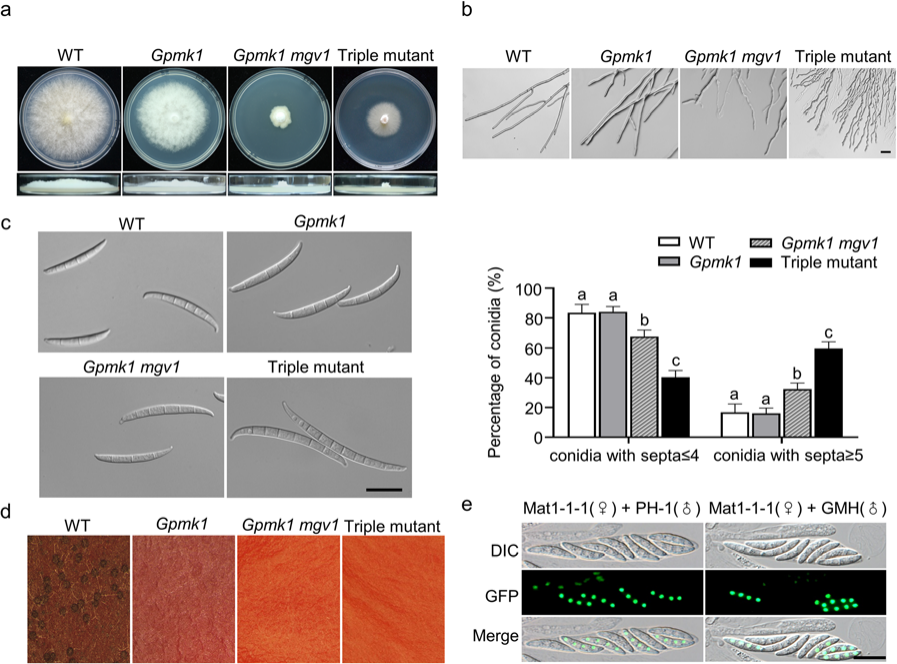
Assay for the roles of MAPKs in hyphal growth, conidiation, and sexual reproduction. **a**. 4-day-old CM cultures and growth rate of the wild type (WT) and *Gpmk1*, *Gpmk1 mgv1* double, and *Gpmk1 mgv1 Fghog1* triple mutants. **b**. Hyphal tip growth and branching patterns of the indicated strains on 1/2 CM plates. Scale bars, 50 μm. **c.** Conidial morphology of the indicated strains. Scale bars, 20 μm. Percentage of conidia with ≤4 and ≥ 5 septa in the indicated strains. **d.** Self-mating cultures of the indicated strains were examined for perithecia and cirrhi at 7 days post-fertilization. **e.** Asci and ascospores from the out-crosses between the *mat1–1* H1-GFP strain (female) and the wild type or triple mutant (male) at 7 dpf were examined by DIC and epifluorescence microscopy. Scale bars, 20 μm. Mean and standard deviation were estimated with data from at least three (*n = 3*) independent biological replicates. Different letters indicate significant differences based on ANOVA analysis followed by Duncan’s multiple range test (*P* = 0.05)

To generate the *Gpmk1 mgv1 Fghog1* triple mutant in *F. graminearum*, we first generated the *Gpmk1 mgv1* double mutant by deleting the *MGV1* gene in the *Gpmk1* mutant (Wang et al. 2011). The resulting double mutant was used to generate the triple mutant by transforming the *FgHOG1* gene replacement construct into the *Gpmk1 mgv1* mutant (Table S1). Deletion of *MGV1* and *FgHOG1* in the *Gpmk1 mgv1 Fghog1* triple mutant was verified by PCR with anchor primers (Table S1, Fig. S1). Three triple mutant strains were identified. All three of them had the same phenotype although only data for one of them were presented below. Unlike the *Gpmk1* and *Fghog1* mutants, the *mgv1* mutant had severe growth defects (Wang et al. 2011). Interestingly, in comparison with the *Gpmk1 mgv1* double mutant, the growth rate was slightly increased in the triple mutant. Nevertheless, the triple mutant still grew slower than the wild type and *Gpmk1* mutant (Fig. 1a; Table 1). Furthermore, aerial hyphae produced by the triple mutant was reduced in surface hydrophobicity, likely caused by deletion of *FgHOG1* (Zheng et al. 2012) (Fig. S2). In comparison with the wild type, the triple mutant produced wavy germ tubes and hyphae (Fig. 1b), which was not observed in the *Gpmk1* and *Gpmk1 mgv1* mutants (Fig. 1b), suggesting an overlapping function of three MAPKs in hyphal growth. These results indicate that deletion of all three MAPKs is not lethal in *F. graminearum* but resulted in defects in hyphal growth and colony surface properties.

**Table 1:** Defects of the *Gmpk1*, *Gpmk1 mgv1* and *Gpmk1 mgv1 Fghog1* mutants in growth, conidiation, and plant infection

	Growth rate (mm/day)^**A**^	Conidiation (× 10^**4**^/ml)^**B**^	Disease index^**C**^
**WT**	9.24 ± 0.36^a^	113.61 ± 10.15^a^	9.9 ± 2.67^a^
***Gpmk1***	7.28 ± 0.15^b^	46.89 ± 7.17^b^	NA
***Gpmk1 mgv1***	2.58 ± 0.15^d^	48.11 ± 18.17^b^	NA
**Triple mutant**	3.72 ± 0.26^c^	47.61 ± 10.80^b^	0^b^

A. Average daily extension in colony radius was measured on CM plates. Mean and standard deviation were calculated from at least three independent measurements

B. Conidiation in 5-day-old CMC cultures

C. Disease index was rated by the number of symptomatic spikelets per wheat head at 14 days post-inoculation (dpi)

Data were analyzed with Duncan’s pairwise comparison. Different letters mark statistically significant differences (*P* = 0.05)

NA. not analyzed

When assayed for conidiation in liquid CMC medium, the *Gpmk1 mgv1 Fghog1* mutant was reduced 2-fold in conidiation compared to the wild type, which was similar to the *Gpmk1* and *Gmpk1 mgv1* mutant (Table 1), indicating the importance of Gpmk1 in conidiation. In addition, conidia of the triple mutant were longer and had more septa than those of the wild type (Fig. 1c). Whereas majority of the wild-type conidia had three or four septa, more than half of the *Gpmk1 mgv1 Fghog1* conidia had five or more septa (Fig. 1c). Conidia of the *Gpmk1 mgv1* double mutant tended to be longer than the wild type and *Gpmk1* mutant, but still shorter and had less septa than those of the triple mutant (Fig. 1c). Because deletion of individual MAPK genes is not known to affect conidium morphology in *F. graminearum* (Hou et al. 2002; Jenczmionka and Schafer 2005; Ren et al. 2019; Wang et al. 2011; Zheng et al. 2012), Gpmk1, Mgv1, and FgHog1 likely hare overlapping or redundant roles in conidiogenesis and conidium morphology.

On self-mating carrot agar cultures, the triple mutant was sterile (Fig. 1d). Whereas abundant perithecia were formed by the wild type at 7 days post-fertilization (dpf), the *Gpmk1 mgv1 Fghog1* mutant failed to produce perithecia under the same conditions (Fig. 1d). We then conducted outcrosses with the *Gpmk1 mgv1 Fghog1* triple mutant to determine whether it is defective in male or female fertility. When the triple mutant was used as the male to fertilize a *mat1–1* H1-GFP strain, fertile perithecia with normal asci and ascospores were observed at 7 dpf (Fig. 1e). Four of the eight ascospores in each ascus had GFP signals in the nucleus, suggesting the segregation of H1-GFP in the progeny (Fig. 1e). Therefore, deletion of all the MAPKs has no effect on male fertility but results in the loss of female fertility in selfing in *F. graminearum*.

### Deletion of all three MAPK genes results in the loss of pathogenicity and DON production

**Fig. 2:**
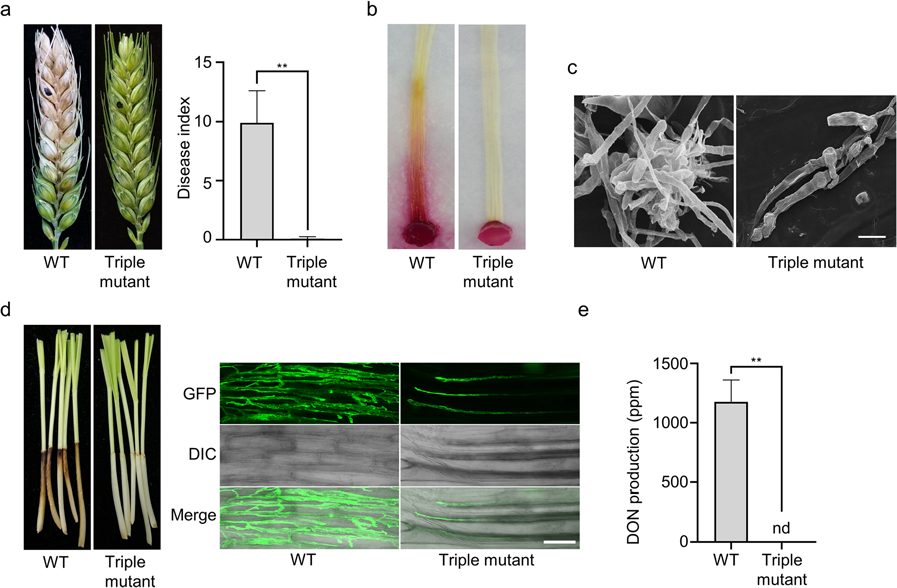
Defects of the *gmpk1 mgv1 Fghog1* triple mutant in plant infection. **a**. Typical wheat heads of cultivar XiaoYan 22 drop-inoculated with conidia of the wild type and *Gpmk1 mgv1 Fghog1* triple mutant were examined for FHB symptoms and measured for disease index at 14 dpi. Black dots mark the inoculated spikelets. **b**. Corn silks inoculated with the wild type and triple mutant were examined at 5 dpi. **c**. Formation of infection cushions on lemma were examined by SEM. Scale bars, 10 μm. **d**. Infected coleoptiles were examined for necrosis and invasive hyphae after staining with Alexa Fluor 488. Scale bars, 50 μm. **e.** DON production in the inoculated wheat kernels sampled at 14 dpi. Mean and standard deviation were estimated with data from at least three (*n = 3*) independent biological replicates. The asterisk indicates significant differences based on Student’s *t*-test. **P < 0.01

In infection assays with flowering wheat heads, the *Gpmk1 mgv1 Fghog1* deletion mutant failed to cause symptoms on the inoculated kernels and were non-pathogenic to wheat (Table 1; Fig. 2a). In infection assays with corn silks, the triple mutant caused very limited discoloration at the inoculation site. Extensive discoloration was observed only in samples inoculated with the wild type (Fig. 2b).

*F. graminearum* is known to form infection cushions for plant penetration. When examined by scanning electron microscopy (SEM), abundant infection cushions were formed by the wild type on wheat lemma at 2 days post-inoculation (dpi) (Fig. 2c). However, typical infection cushions were rarely formed by the triple mutant (Fig. 2c). In infection assays with wheat coleoptiles, extensive invasive growth and necrosis were only observed in samples inoculated with the wild type. In samples inoculated with *Gpmk1 mgv1 Fghog1* conidia, invasive hyphae were often restricted to the initial penetrated coleoptile cells (Fig. 2d). Extensive spreading of invasive hyphae and necrosis beyond the wounding inoculation site were rarely observed in samples inoculated with the triple mutant, suggesting the importance of these MAPKs during infection cushion formation and infectious growth.

Because DON is an important virulence factor, we assayed DON production during plant infection. In wheat kernels inoculated with the wild type and collected at 14 dpi, over 1000 ppm DON was detected. Under the same conditions, DON was not detectable in samples inoculated with the *Gpmk1 mgv1 Fghog1* mutant (Fig. 2e). Taken together, these results indicated that plant infection and DON biosynthesis are blocked when all three MAPKs are deleted.

### The triple mutant is hypersensitive to cell wall, osmotic, and oxidative stresses

**Fig. 3:**
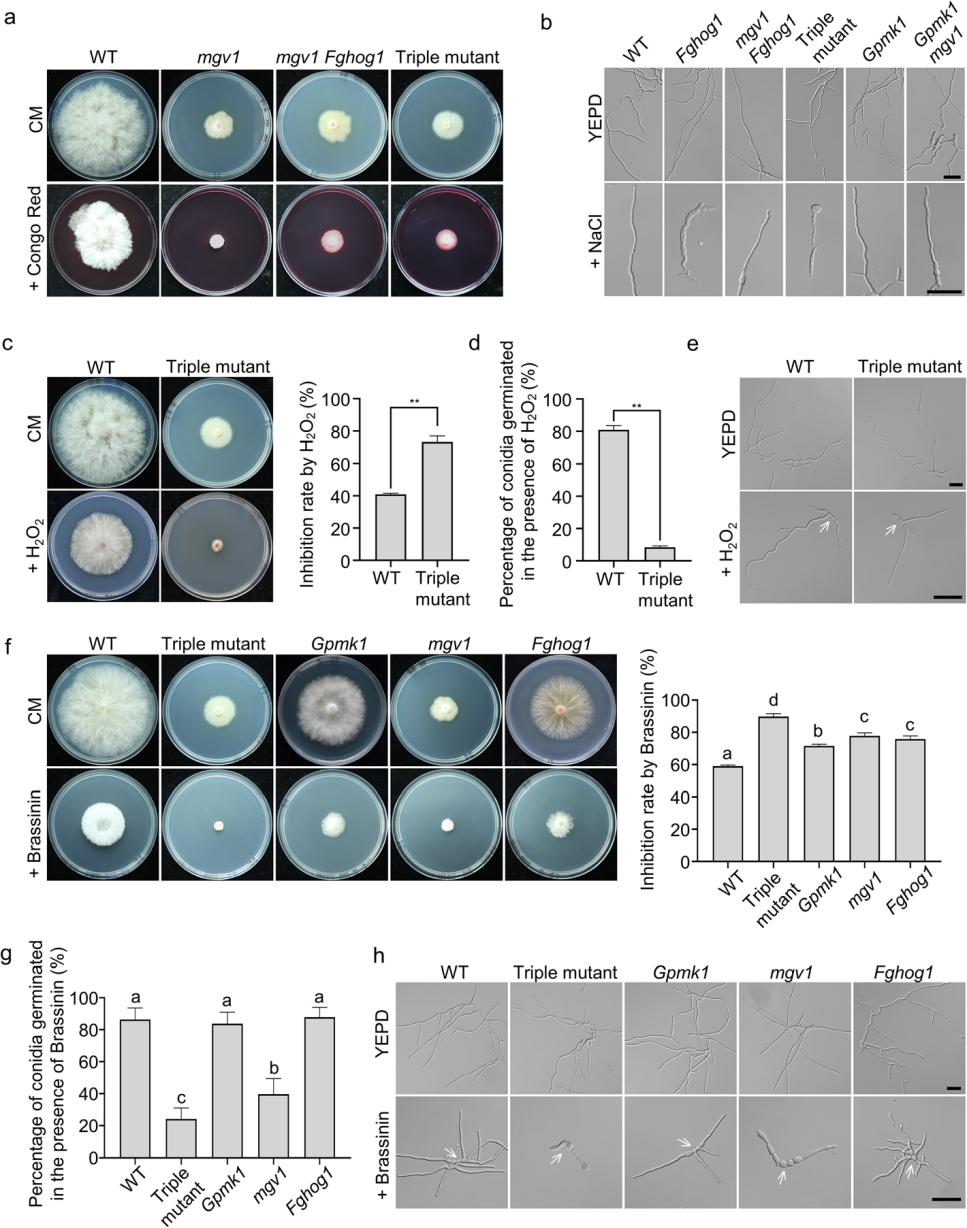
The *gmpk1 mgv1 Fghog1* triple mutant were defective in responses to abiotic stresses. **a**. Cultures of the indicated strains grown on regular CM and CM medium with 200 μg/ml Congo red. **b**. Conidia of the indicated strains were incubated for 12 h in YEPD with or without 0.7 M NaCl. Scale bars, 50 μm. **c**. 4-day-old cultures of the wild type and triple mutant grown on CM with or without 0.05% H_2_O_2_. The inhibition rate by H_2_O_2_ is estimated as the percentage of reduction in colony diameters on CM with H_2_O_2_ in comparison with regular CM. **d**. Percentage of conidia germinated in YEPD medium after incubation for 6 h. **e**. Conidia of the wild type and *Gpmk1 mgv1 Fghog1* triple mutant were incubated for 12 h in YEPD with or without 0.005% H_2_O_2_. Scale bars, 50 μm. Conidia were indicated by arrows. **f.** CM cultures of the indicated strains with or without 250 μM brassinin. The inhibition rate by brassinin on growth in the indicated strains were estimated with colony diameters measured with 4-day-old cultures. **g**. Percentage of conidia germinated in YEPD medium after incubation for 6 h. **h.** Conidia of the labelled strains incubated in YEPD with or without 250 μM brassinin for 24 h. Scale bars, 50 μm. Conidia were indicated by arrows. Mean and standard deviation were estimated with data from at least three (*n = 3*) independent biological replicates. Different letters indicate significant differences based on ANOVA analysis followed by Duncan’s multiple range test (*P* = 0.05). The asterisk indicates significant differences based on Student’s *t*-test. **P < 0.01

The Mgv1 MAPK is known to have a conserved role in cell wall integrity (CWI) and deletion of *FgHOG1* partially suppress the hypersensitivity of *mgv1* mutant to cell wall stress (Ren et al. 2019). When cultured on CM medium with 200 μg/ml Congo red (CR), a commonly used cell wall stressor, the *Gpmk1 mgv1 Fghog1* triple mutant displayed similar sensitivity as the *mgv1 Fghog1* double mutant, but grew better than the *mgv1* mutant (Fig. 3a). Therefore, Gpmk1 is likely dispensable for cell wall stress responses when Mgv1 and FgHog1 are deleted.

In general, fungal osmotic regulation is mainly regulated by the well-conserved HOG MAPK pathway although the other two MAPKs may have minor effects in regulating responses to hyperosmotic stresses in some fungi (Zhang et al. 2021). In the presence of 0.7 M NaCl, germ tubes had subapical swelling in the *Fghog1* mutant and apical swelling in the triple mutant. Nevertheless, irregular swellings were not observed in the *Gpmk1*, *Gpmk1 mgv1*, and *mgv1 Fghog1* mutants (Fig. 3b). These results indicated that Gpmk1 is not important for osmoregulation in the wild type but it may have opposite roles with Mgv1 on responses to hyperosmotic stress in the absence of FgHog1.

In comparison with the wild type, the triple MAPK mutant also displayed increased sensitivity to oxidative stress and only formed compact colonies with limited growth in the presence of 0.05% H_2_O_2_ (Fig. 3c). Over 90% of *Gpmk1 mgv1 Fghog1* conidia failed to germinate after incubation for 6 h in the presence of H_2_O_2_, which had only a minor effect on conidium germination in the wild type (Fig. 3d). When cultured for 12 h in YEPD liquid medium with H_2_O_2_, more severe defects in germ tube growth were observed in the triple mutant than that in the wild type (Fig. 3e).

Brassinin is a phytoalexin with antifungal activities against a number of fungal pathogens (Sellam et al., 2007). The *Gpmk1 mgv1 Fghog1* triple mutant also was hypersensitive to brassinin (Fig. 3f). The presence of 250 μM brassinin inhibited not only colonial growth but also conidium germination significantly in the triple mutant in comparison with the wild type (Fig. 3f; g). Deletion of *GPMK1*, *MGV1,* or *FgHOG1* individually also resulted in hypersensitivity to brassinin in colonial growth, but the *Gpmk1* mutant had a higher level of tolerance against brassinin than the other two MAPK mutants (Fig. 3f). In contrast, the *mgv1* mutant but not the other two MAPK mutants had conidium germination defects in the presence of brassinin although less severe than the triple mutant (Fig. 3g). Microscopic examination revealed a severe defect of germ tube growth in the triple mutant when cultured in liquid YEPD with brassinin (Fig. 3g). Germ tubes of the *Gpmk1*, *mgv1,* and *Fghog1* mutant also tended to be shorter than those of the wild type in presence of brassinin, and the *mgv1* mutant exhibited the highest sensitivity to brassinin (Fig. 3h). Therefore, Mgv1 plays a major role in regulating responses to brassinin although the Gpmk1 and FgHog1 MAPKs also have minor effects.

### Mgv1 MAPK plays a major role in the fungal-bacterial interaction

**Fig. 4:**
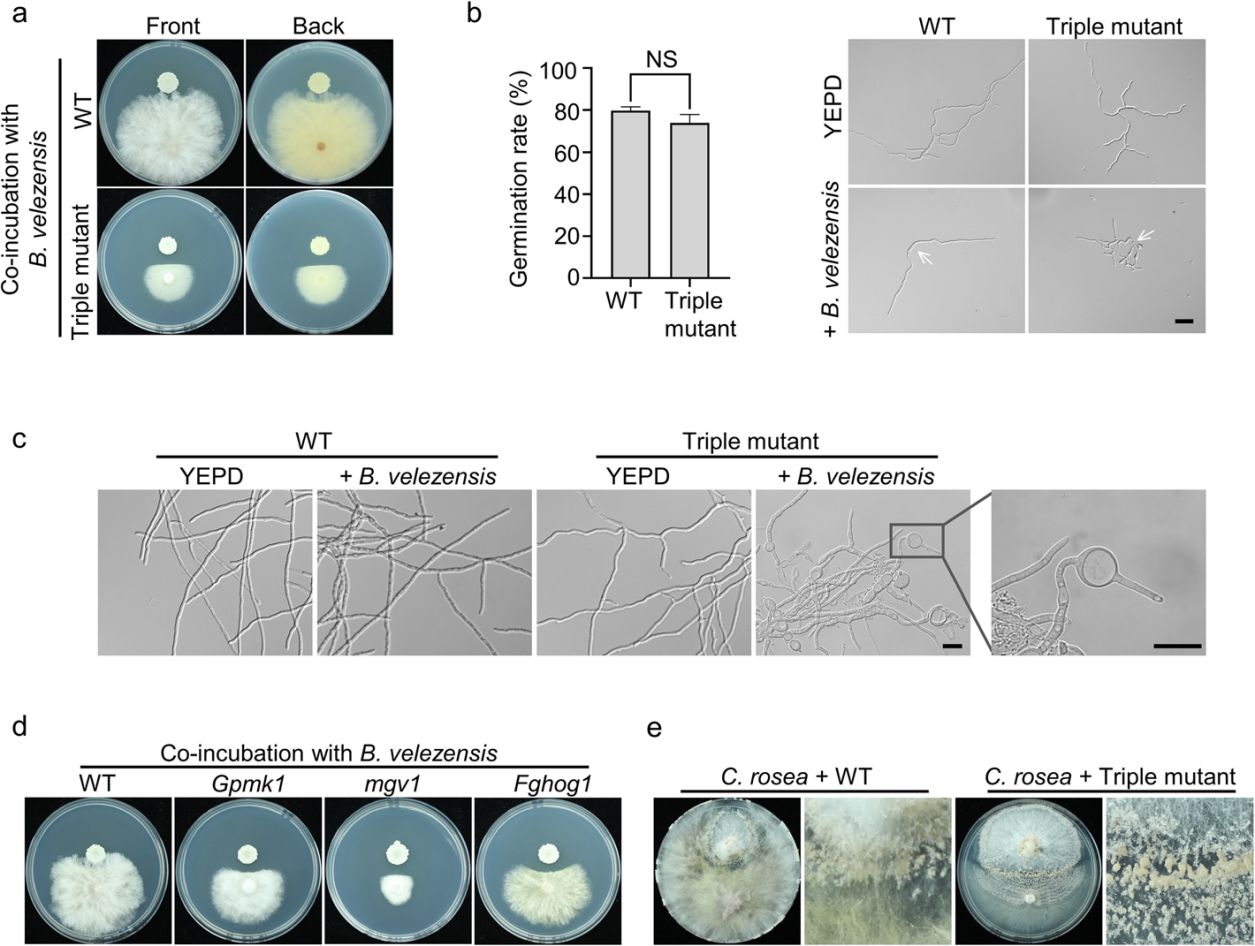
Roles of MAPKs in bacterial-fungal and fungal-fungal interactions. **a**. Antagonistic activity of *B. velezensis* towards the wild type and *Gpmk1 mgv1 Fghog1* triple mutant. **b**. The inhibitory effects of *B. velezensis* on conidium germination (6 h) and germ tube growth (12 h). Scale bars, 20 μm. **c**. The formation of swollen hyphal compartments in the triple mutant stimulated by *B. velezensis*. Scale bars, 50 μm. Conidia were indicated by arrows. **d.** Antagonistic activity of *B. velezensis* towards the wild type and *Gpmk1*, *mgv1,* and *Fghog1* mutants. **e.** 16-day-old CM confrontation-cultures of *C. rosea* (top) with the wild type or triple mutant of *F. graminearum* (bottom) with the initial contact zone amplified on the right to show stimulated conidiation in *C. rosea*. Mean and standard deviation were estimated with data from at least three (*n = 3*) independent biological replicates. NS indicates no significant differences based on Student’s *t*-test

Because MAPKs are likely involved in bacterial-fungal interactions in *F. graminearum* (Zhang et al. 2021), we then assayed the effect of co-incubation with *Bacillus velezensis* strain ZQT, a biocontrol agent that inhibits pathogen growth as well as induces systemic resistance in plants (Fira et al., 2018). In antagonistic tests, the *Gpmk1 mgv1 Fghog1* triple deletion mutant showed increased sensitivity to *B. velezensis* in comparison with the wild type (Fig. 4a). Treatments with *B. velezensis* resulted in highly vacuolated conidia and shorter germ tubes that appeared to swell at the tip in the triple mutant, which was rare in the wild type (Fig. 4b). In the presence of *B. velezensis*, abundant swollen hyphal compartments were stimulated in the triple mutant but similar irregular swellings were rarely observed in the wild type (Fig. 4c). These results suggest that *B. velezensis* interferes with the maintenance of polarity at germ tube or hyphal tips.

We then compared the sensitivities of the mutants deleted of individual MAPK genes to *B. velezensis*. Although all of them had elevated sensitivity, the *mgv1* mutant was more sensitive to *B. velezensis* than the *Gpmk1* and *Fghog1* mutants (Fig. 4d). Interestingly, the *mgv1* mutant appeared to be more sensitive to *B. velezensis* than the *Gpmk1 mgv1 Fghog1* triple mutant (Fig. 4a; 4d), suggesting that deletion of *GPMK1* or *FgHOG1* may partially rescues its defects in fungal-bacterial interactions.

### **The triple mutant is defective in fungal-fungal interactions with***Clonostachys rosea*

Because MAPKs have also been implicated in mycoparasitic interactions between fungi (Zhang et al. 2021), we assayed the interactions of MAPK mutants with *C. rosea*, a biocontrol agent against fungal pathogens (Sun et al., 2020a). In confrontation assays, the wild-type strain PH-1 of *F. graminearum* grew faster than *C. rosea* strain CanS41 (Fig. 4e). Although there was a small inhibition zone at the edge of *C. rosea* colonies that were surrounded by PH-1 hyphae, we did not observe growth of one fungus over the other (Fig. 4e). Interestingly, it appears that conidiation was stimulated in *C. rosea* at the edge of its colonies by PH-1 (Fig. 4e). Under the same conditions, no clear inhibition zone was observed between *C. rosea* and the *Gpmk1 mgv1 Fghog1* triple mutant. In fact, *C. rosea* grew over colonies of the triple mutant after co-incubation for 16 days and had three distinct zones. Conidiation in *C. rosea* was stimulated in the initial confrontation zone and the zone with overlapping growth with the triple mutant (Fig. 4e), likely due to antagonistic interactions with *F. graminearum*. In the zone beyond the triple mutant colony, hyphal growth became sparse and no stimulated conidiation was visible in *C. rosea* (Fig. 4e). These results suggest that the wild type, but not the triple mutant, is able to defend against mycoparasitic *C. rosea*. The defense response against *C. rosea* must be significantly weakened in the mutant deleted of all three MAPKs.

### Lack of MAPKs affects the expression of many ABC and MFS transporter genes

**Fig. 5:**
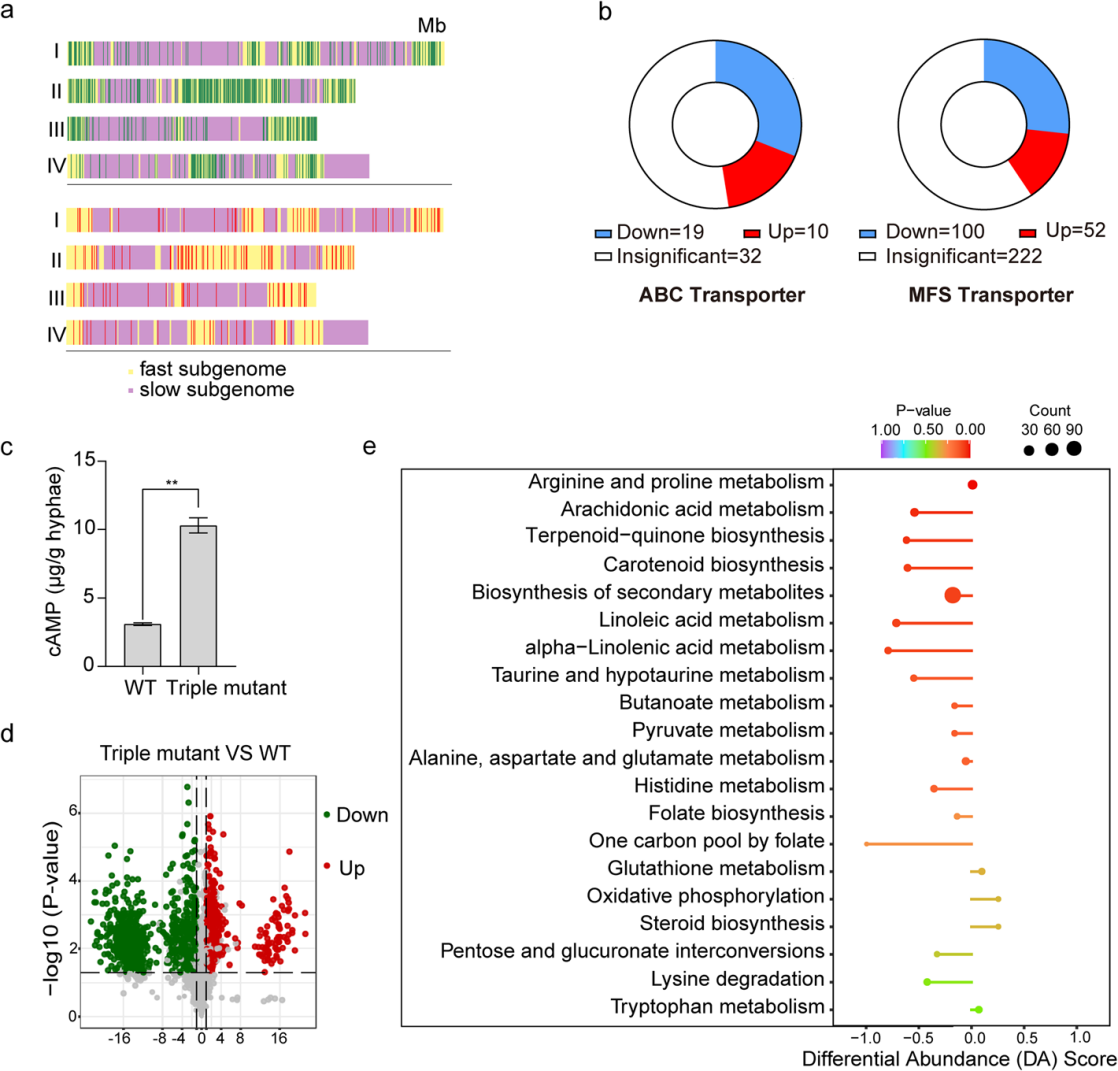
RNA-seq analysis and metabolome profiling of the wild type and *Gpmk1 mgv1 Fghog1* triple mutant. **a**. Distribution of up-regulated (red) and down-regulated (green) DEGs in the triple mutant on chromosomes I to IV. Each vertical line represents the chromosomal position of a specific DEG. Yellow and purple region represent the fast and slow subgenomic regions of *F. graminearum*, respectively. **b**. Expression patterns of all the predicted ABC and MFS transporter genes in the wild type and triple mutant. Up and down indicate the ones that were up- or down-regulated in the triple mutant. **c.** The intracellular cAMP level in the wild type and triple mutant. **d**. A volcano plot of the 407 and 806 metabolites that were significantly increased (red dots) and decreased (green dots), respectively, in the triple mutant in comparison with the wild type. **e**. KEGG enrichment analysis of differentially accumulated metabolites related to listed metabolic pathways. The differential abundance (DA) score reflects the quantitative change of metabolites in the triple mutant (−, decreased; + increased) compared to the wild type. The sizes of the circles represent the number of differentially accumulated metabolites in each metabolism pathway. Distance between the circle and the center point represent the range of variation

To analyze the effects of loss of all MAPKs, we performed RNA-seq with the wild type and *Gpmk1 mgv1 Fghog1* triple mutant with three biological replicates for each. In comparison with the wild type, 1469 genes and 2203 genes were up- and down-regulated over two-fold in the triple mutant, respectively (Table S2; Fig. S3). Interestingly, most of these differentially expressed genes (DEGs) were in the fast evolving subgenome associated with heterochromatins (Fig. 5a), indicating that genes regulated by these three MAPKs were subjected to fast evolution.

Based on Gene Ontology (GO)-enrichment analysis, the top 500 up-regulated DEGs in the triple mutant were not enriched in any biological process. However, the top 500 down-regulated DEGs were significantly enriched for transmembrane transport, carbohydrate transport, interspecies interactions between organisms, and oxidation-reduction process (Fig. S4).

Because transport-related genes are enriched in DEGs based on GO analysis, we examined the expression profiles of all the predicted ATP-binding cassette (ABC) and major facilitator superfamily (MFS) transporter genes in *F. graminearum*. In comparison with the wild type, 10 up-regulated and 19 down-regulated ABC transporter genes were identified in the triple mutant (Fig. 5b). For differentially expressed MFS transporter genes, 52 had increased transcription levels in the triple mutant whereas deletion of all three MAPKs reduced the expression of 100 MFS transporter genes (Fig. 5b). These ABC and MFS transporter genes are likely under transcriptional regulation by three MAPK pathways.

Interestingly, the expression level of the *FAC1* adenylate cyclase gene was higher in the triple MAPK mutant than the wild type (Table S2). When intracellular cAMP level was measured, the triple mutant also showed an elevated cAMP level in comparison with the wild type (Fig. 5c). These results indicated that deletion of all three MAPK genes results in the overstimulation of the cAMP-PKA signaling in *F. graminearum*. In contrast, three MEKK (*FST11*, *BCK1* and *SSK2*) and three MEK (*FST7*, *MKK2* and *PBS2*) genes had no significant changes in their expression levels in the triple mutant. The expression levels of genes encoding the Gα, Gβ, and Gγ proteins also were normal in the tripe mutant, suggesting that the expression of well-conserved upstream G-proteins, MEKKs, and MEKs are not affected by deletion of these three MAPKs. In addition, deletion of three MAPKs resulted in no significant effect on the expression levels of the well-conserved *STE12*, *MCM1*, *SWI6*, *RLM1*, and *ATF1* orthologs that are downstream transcription factors of MAPK signaling in the budding yeast (Jiang et al., 2018a; Zhang et al., 2021).

### Significant changes in metabolic profiles in the ***Gpmk1 mgv1 Fghog1*** triple mutant

To assay the effects of no MAPK signaling on metabolism, hyphae of the wild type and *Gpmk1 mgv1 Fghog1* triple mutant were collected for widely-targeted metabolomics analysis. All three biological replicates were grouped together, indicating a high-reliability of the metabolome data (Fig. S5). A clear separation between the wild type and triple mutant were detected, suggesting the metabolite profiles in these two samples are obviously distinct (Fig. S5). A total of 2230 metabolites were identified in the metabolome of *F. graminearum*, including 1213 differentially accumulated metabolites (over two-fold changes) and 1017 metabolites with no significantly changes between the wild type and triple mutant (Fig. 5d). Among the differentially accumulated metabolites, 407 metabolites were up-regulated while 806 metabolites were down-regulated in the triple mutant (Fig. 5d). The top enriched KEGG terms among the differentially accumulated metabolites were related to arachidonic acid, linoleic acid, and alpha-linolenic acid metabolisms, and biosynthesis of terpenoid-quinone and carotenoid (Fig. 5e). Interestingly, linoleic acid and α-linolenic acid were previously reported to have antibacterial (Kusumah et al., 2020) and antifungal activities (Walters et al., 2004), which may contribute to the defects of the triple mutant in fungal-bacterial and fungal-fungal interactions.

## Discussion

The roles of individual MAPKs in regulating developmental and infection processes, as well as stress responses have been characterized in a variety of fungal pathogens that differ in host ranges and infection mechanisms (Jiang et al. 2018a). However, the effect of deletion of all three MAPKs in fungal growth, differentiation, and response to biotic and abiotic stresses has not been reported in any plant pathogenic fungi or filamentous ascomycetes. In this study, we found that the *Gpmk1 mgv1 Fghog1* triple mutant was viable but displayed pleiotropic defects that differ from the *mgv1*, *Gpmk1*, and *Fghog1* single mutants (Fig. S6). For vegetative growth, the *mgv1* mutant displays severe growth defects but the *Gpmk1* and *Fghog1* mutants have only a minor reduction in growth rate (Hou et al. 2002; Wang et al. 2011; Zheng et al. 2012). Our earlier study has showed that null mutations in *FgHOG1* partially rescued growth rate of the *mgv1* mutant (Ren et al. 2019). In this study, we showed that the triple mutant grew faster than the *mgv1* mutant, indicating that in the absence of *GPMK1*, *FgHOG1* is also suppressive to the growth defect caused by *MGV1* deletion. However, whereas the *Gpmk1* mutant had an approximately 21% reduction in growth rate compared to the wild type, deletion of *GPMK1* in the *mgv1 Fghog1* double mutant resulted in a 31% decrease in growth rate (Table 1). Therefore, Gpmk1 likely contributes to the suppressive effect of *FgHOG1* deletion on *mgv1*. Gpmk1 may have overlapping functions with either Mgv1 or FgHog1 in vegetative growth. In addition, the *Gpmk1 mgv1 Fghog1* triple mutant was reduced in conidiation but had abnormal conidium morphology. Because Mgv1 is dispensable for conidiation (Hou et al. 2002), the defect of the triple mutant in conidiation is likely due to deletion of *GPMK1* and *FgHOG1*, which is known to affect conidiation (Jenczmionka et al. 2003; Zheng et al. 2012). However, production of morphologically abnormal conidia has not been reported in any of the MAPK mutants (Hou et al. 2002; Jenczmionka and Schafer 2005; Ren et al. 2019; Wang et al. 2011; Zheng et al. 2012). It is likely that deletion of both *GPMK1* and *FgHOG1* is responsible for the production of longer conidia with more septa than the wild-type conidia in the triple mutant. By examining the expression levels of *CON1*, *CON2*, *COM1*, and *HTF1* (transcription factors known to affect conidiation in *F. graminearum*) in our RNA-seq data, *CON1* expression was affected (down-regulated 5-fold) in the triple mutant, likely related to the defects in conidiogenesis. In addition, deletion of three MPAKs may increase the intracellular cAMP level, which in turn activates the cAMP-PKA signaling pathway. The hyper-activation of cAMP-signaling may partially rescue some defects of the MAPK triple mutant but result in additional defects in conidiogenesis.

Because all three MAPKs are important for plant infection, it is not surprising that the triple mutant was non-pathogenic in wheat head and corn silk infection assays (Fig. S6). In *M. oryzae* and several other plant pathogens, the *PMK1* and *MPS1* MAPKs are known to regulate the formation of infection structures, penetration, and growth of invasive hyphae in infected plant tissues (Jiang et al. 2018a). In *F. graminearum*, the triple mutant was defective in infection cushion formation, plant penetration, and invasive growth. Although *GPMK1* and *MGV1* MAPKs as well as their upstream kinases are known to be essential for pathogenesis in earlier studies (Hou et al. 2002; Wang et al. 2011; Yun et al. 2014), their regulatory roles in infection cushion formation and invasive growth have not been characterized. Nevertheless, the *GIV1* GPCR gene that appears to function upstream of Gpmk1 is involved in regulating the development of infection cushions in *F. graminearum* (Jiang et al. 2019). DON is an important virulence factor that enables the fungus to spread from infected florets to the wheat rachis and other florets on the same flowering head (Bai et al., 2002; Jansen et al., 2005). The defect of the triple mutant in DON production may contribute to its defect in pathogenesis. In addition, we showed that the triple mutant was hypersensitive to phytoalexin brassinin. In *Alternaria brassicicola*, both AbSlt2 (Mgv1 ortholog) and AbHog1 are activated by camalexin, an indolic phytoalexin structurally related to brassinin (Joubert et al., 2011). In *F. graminearum*, the *Gpmk1, mgv1* and *Fghog1* mutants were all sensitive to brassinin, suggesting that these MAPKs have overlapping functions in regulating cellular responses to phytoalexins. Furthermore, our RNA-seq data showed that differentially expressed genes (DEGs) in the triple mutant were enriched in the fast evolving subgenome, which is under the control of heterochromatin and likely contains many genes important for adaption and infection (Wang et al., 2017). Some of these DEGs regulated by MAPK pathways likely play important roles in different infection processes or fungal-plant interactions.

In filamentous fungi, the CWI and HOG MAPK pathways are generally considered to be the major regulators of responses to cell wall stress and high osmolarity, respectively. While deletion of *FgHOG1* was suppressive to the cell wall and growth defects of the *mgv1* mutant, deletion of *MGV1* slightly alleviated the hyperosmotic sensitivity of the *Fghog1* mutant (Ren et al. 2019), suggesting an opposite role of *MGV1* and *FgHog1* in response to abiotic stressors. Nevertheless, the role of Gpmk1 and its relationship with the other two MAPKs (Mgv1 and FgHog1) in stress response are not clear in *F. graminearum*. Because the *Gpmk1 mgv1 FgHog1* triple mutant displayed similar sensitivity to CR as the *mgv1 Fghog1* double mutant, indicating that Gpmk1 fails to function as a MAPK important for regulating responses to cell wall stress when *MGV1* and *FgHOG1* are deleted. Indeed, the role of Gpmk1 orthologs in cell wall integrity varies among different fungi. Whereas deletion of ChMK1 resulted in hypersensitivity to CR in *Colletotrichun higginsianum* (Wei et al., 2016), the *Cfpmk1* mutant was more tolerant against CR than the wild type in *C. fructicola* (Liang et al., 2019). It will be important to further characterize the role of Gpmk1 in cell wall integrity and its relationship with Mgv1 and FgHog1 in *F. graminearum*. In the presence of osmotic stress, abnormal germ tubes with irregular apical and subapical swelling were observed in the triple mutant and *Fghog1* mutant, but not in the *mgv1 Fghog1* double mutant. It is likely that Mgv1 and Gpmk1 have opposite roles on responses to hyperosmotic stress in the absence of FgHog1. Interestingly, the *Gpmk1* mutant and *Gpmk1 mgv1* double mutant were also normal in germ tube morphology, indicating that Gmpk1 only plays a minor role in hyperosmotic sensitivity in the absence of FgHog1. Therefore, the increased phosphorylation level and over-activation of Gpmk1 in the *mgv1 Fghog1* double mutant (Ren et al. 2019) may contribute to the suppressive effects of *FgHOG1* deletion on the *mgv1* mutant.

Plant pathogenic fungi such as *F. graminearum* have to interact and compete with other microbes for survival on plant debris and during plant infection (Chen et al., 2018; Zhang et al. 2021). In comparison with the roles of MAPKs in response to abiotic stresses, less is known about their functions in interactions with bacteria and other fungi. In this study, we found that the triple mutant was hypersensitive to *B. velezensis* (Jiang et al., 2018b). *MGV1* likely plays a major role in regulating the interaction of *F. graminearum* with *B. velezensis* because the *mgv1* mutant appeared to be more sensitive than the triple mutant (Fig. S6). Although the *Gpmk1* and *Fghog1* mutants were slightly increased in sensitivity, deletion of *FgHOG1* and *GPMK1* in the *mgv1* mutant increased its resistance against *B. velezensis*, suggesting possible crosstalk among these MAPKs in regulating fungal-bacterial interactions. Limited studies in other fungi (such as *S. cerevisiae*, *V. dahlia* and *Rhizopus microspores*) have showed that MAPKs can be activated by microbe-associated molecular patterns (MAMPs) or interactions with bacteria (Han et al., 2015; Lastovetsky et al., 2016; Marques et al., 2006). In *F. graminearum*, the phosphorylation of Mgv1 was inhibited by HopAI, an effector from *Pseudomonas syringae* (Zhang et al., 2017). Similar to their plant and animal counterparts, filamentous fungi have Nod-like immune receptors (NLRs) for signal recognition and activation of downstream targets (Uehling et al., 2017). *F. graminearum* has over 60 predicted NLR genes and some of them may be involved in recognizing MAMPS and fungal-bacterial interactions.

The endophytic fungus *C. rosea* is a well-known biological control agent against diverse phytopathogenic fungi (Demissie et al., 2018). In confrontation assays, the wild type *F. graminearum* was able to defend against mycoparasitic hyphae and grew surrounding *C. rosea* colonies with a small inhibition zone in their antagonistic interactions. Deletion of all three MAPKs appeared to compromise its ability to defend against *C. rosea*, which grew over the triple mutant and often resulted in hyphal lysis in *F. graminearum* after incubation for 16 days (Fig. S6). As a potent inhibitor of protein synthesis in eukaryotic organisms by binding to the large ribosome subunit, DON is an important virulence factor during plant infection and may also play an important role in antagonistic interactions with other fungi (Chen et al., 2019). The triple mutant was blocked in DON production, which may contribute to its defects in biotic interactions with *C. rosea*. In addition, the triple mutant had defects in cell wall integrity. A weakened cell wall may also affect fungal-fungal interactions. Nevertheless, the triple mutant still stimulated conidiation in *C. resea*, suggesting that it may still produce other antagonistic compounds. Although MAPKs are known to be related to mycoparasitism in mycoparasitic fungi (Mukherjee et al., 2003; Reithner et al., 2007; Sun et al., 2020b), their roles in fungal-fungal interactions have not been reported in any plant pathogenic fungi. Therefore, it is important to further characterize the roles of MAPK pathways and their upstream receptors in antagonistic fungal-fungal interactions in *F. graminearum* and other fungal pathogens.

## Materials & methods

### Fungal strains and culture conditions

The wild-type *F. graminearum* strain PH-1 and MAPK mutants generated in this study were routinely cultured on potato dextrose agar (PDA). Complete medium (CM) cultures grown at 25 °C were used for assaying growth rate and colony morphology (Wang et al. 2011). Conidiation was assayed with 5-day-old carboxymethylcellulose (CMC) cultures as described (Hou et al. 2002). For sexual reproduction, aerial hyphae of 7-day-old carrot agar cultures were pressed down and incubated at 25 °C under black light as described (Jiang et al., 2016). Protoplast preparation and polyethylene glycol (PEG)-mediated transformation were performed as described (Hou et al. 2002). Hygromycin B, geneticin, and fludioxonil were added to the final concentration at 300, 400 and 10 μg/ml, respectively, for transformant selection.

### Generation of the ***Gpmk1 mgv1 Fghog1*triple** deletion mutant

To generate the *Gpmk1 mgv1* double mutant, the 982 kb upstream and 690 kb downstream flanking sequences of *MGV1* were amplified with primer pairs MGV1F/2R, MGV3F/4R (Table S1), respectively. The resulting PCR products were purified and connected to the neomycin resistance gene cassette by overlapping PCR and transformed into protoplasts of the *Gpmk1* mutant as described (Wang et al. 2011; Zhou et al., 2011). Transformants resistant to both hygromycin and geneticin were confirmed by PCR. To generate the triple mutant, the flanking sequences of *FgHOG1* were amplified with primer pairs HOG1F/2R and HOG3F/4R (Table S1), and then connected with the neomycin resistance gene cassette by overlapping PCR. The resulting PCR product was transformed into protoplasts of the *Gpmk1 mgv1* double mutant. Transformants resistant to hygromycin, geneticin, and fludioxonil were verified by PCR for the deletion of *FgHOG1* (Ren et al. 2019).

### Assays for defects in responses to abiotic stresses

The final concentration of 200 μg/ml CR, 0.05% H_2_O_2_ or 250 μM brassinin was added to CM to assay for colony growth at 25 °C as described (Wang et al. 2011). Colony morphology was examined and photographed after incubation for 4 days. To assay for conidium germination and germ tube growth, the final concentration of 0.7 M NaCl, 0.005% H_2_O_2_ or 250 μM brassinin was added to freshly harvested conidia (10^6^ spores ml^− 1^) resuspended in YEPD medium. After incubation at 25 °C for 6 h, germination rates were counted and analyzed. After incubation at 25 °C for 12 h or 24 h, germlings were examined with an Olympus BX-51 microscope. Each experiment was repeated at least three times independently.

### Plant infection and DON biosynthesis assays

Conidia of PH-1 and the triple mutant were harvested from 5-day-old CMC cultures and resuspended to 10^5^ spores ml^− 1^ in sterile double-distilled water (DDW). Wheat heads of 6-week-old cultivar XiaoYan 22 were inoculated with 10 μl of conidium suspensions at the fifth spikelet from the base (Gale et al., 2007). Spikelets with typical FHB symptoms were examined at 14 dpi to estimate the disease index as described (Ding et al. 2009). Mean and standard deviation of the disease index were estimated with data from three independent replicates with at least ten wheat heads examined in each replicate. DON production in the inoculated spikelets sampled at 14 dpi was assayed by GCMS-QP2010 with AOC-20i autoinjector (Hu et al., 2014).

To assay the formation of infection cushions, wheat lemmas inoculated with *F. graminearum* conidia were sampled at 2 dpi, fixed with 4% (vol/vol) glutaraldehyde, and coated with gold-palladium before examination with a JEOL 6360 scanning electron microscope as described (Boenisch and Schafer 2011). To assay for infectious growth, the top 1–2 mm portion of wheat coleoptiles was excised and inoculated with 2 μl of freshly prepared conidium suspensions (10^5^ spores ml^− 1^) over the wound sites (Zhang et al., 2012). After culture at 25 °C with a 12 h light/12 h dark photoperiod for two days, inoculated seedlings were stained with Alexa Fluor 488 and examined for invasive hyphae with a Olympus FV3000 confocal microscope (Jiang et al. 2019).

### Assays for fungal-bacterial and fungal-fungal interaction

To assay fungal-bacterial interactions, *B. velezensis* strain ZQT (Chen et al. 2018) and *F. graminearum* strains (PH-1 and the MAPK mutants) were inoculated 2.5 cm apart on the opposite side of CM medium. After incubation at 25 °C for 3 days, inhibition of colonial growth was examined. To assay its inhibitory effects on conidium germination and hyphal growth, 3 ml *B. velezensis* (OD600 = 1.0) was mixed with fungal conidia and then resuspended in liquid YEPD (10^5^ spores ml^− 1^). After incubation at 25 °C for 6 h or 12 h, germination and germlings were examined with an Olympus BX-51 microscope. Tip or intercalary swelling was observed after 24 h incubation. Each experiment was repeated at least three times independently.

To assay for fungal-fungal interactions, *C. rosea* strain CanS41 (Demissie et al. 2018) and *F. graminearum* strains were inoculated 3.5 cm apart on CM plates and incubated 25 °C. Growth of both fungi and their antagonistic interactions were observed daily or periodically for up to 16 days.

### RNA-seq and metabolome analyses

Total RNAs isolated from aerial hyphae cultured on 5-day-old CM medium were used for sequencing with Illumina HiSeq 2500 at Novogene Bioinformatics Technology (Beijing). RNA-seq reads were mapped to the PH-1 reference genome using HISAT2 with its two-step algorithm (Kim et al., 2015). RNA-seq data were deposited in the NCBI SRA database under accession numbers SRR16364330–16364335. The number of reads (counts) aligned to each gene was calculated by FeatureCounts. Differentially expressed genes were identified by read count analysis with edgeRun using TMM normalization as described (Dimont et al., 2015). GO annotation was carried out with Blast2GO and GO enrichment analysis was performed by the parent-child union method with Benjamini-Hochberg correction as developed in Ontologizer (Conesa et al., 2005).

Hyphae freshly harvested from 5-day-old CM cultures were used for metabolite profile analysis at Wuhan MetWare Biotechnology Co., Ltd. The freeze-dried samples were ground to a uniform powder prior to metabolomics analyses. The resulting samples were used for metabolite profiling with a liquid chromatography-electrospray ionization-tandem mass spectrometry (LC-ESI-MS/MS) system (HPLC, Shim-pack UFLC SHIMADZU CBM30A system; MS, Applied Biosystems 6500 Q TRAP) as described (Sun et al., 2021). Metabolite quantification was performed with multiple reaction monitoring (MRM) in triple-quadrupole mass spectrometry (Chen et al., 2013). Metabolites were identified by comparing the fragmentation patterns, retention times, and accurate m/z values with the standards in the MetWare and public databases. The relative importance of each metabolite to the PLS-DA model was checked with the parameter called variable importance in projection (VIP). Metabolites with VIP ≥ 1 and fold change ≥2 were considered as differential metabolites for group discrimination (Zhang et al., 2020). KEGG enrichment analysis of differentially accumulated metabolites was performed using KOBAS 2.0 (Xie et al., 2011).

### Data accessibility statement

The data that support the findings of this study are available from the corresponding authors upon request. RNA-seq data generated in this study are accessible under accession numbers SRR16364330–16364335.

## Supplementary Information

**Additional file 1: Fig. S1.** Generation of triple deletion mutants of MAPKs. Lane Up and lane Down showed the occurrence of homologous recombination at the upstream and downstream flanking sequences of the labelled MAPK gene, respectively. Lane T shows the deletion of labelled MAPK genes. Lane H or G showed the amplification of selectable marker genes.

**Additional file 2: Fig. S2.** Surface hydrophobicity assays with the wild type and triple mutant. Photos were taken 15 min after placing droplets of 20 μl red ink on the colony surface.

**Additional file 3: Fig. S3.** A volcano plot of the 1469 up-regulated (red dots) and 2203 down-regulated (green dots) DEGs in *Gpmk1 mgv1 Fghog1* triple mutant compared to the wild type.

**Additional file 4: Fig. S4.** GO enrichment analysis of the down-regulated DEGS in the triple mutant. BP, Biological Process; CC, Cellular Components; MF, Molecular Function.

**Additional file 5: Fig. S5.** Multidimensional scaling plot of the metabolomic profiles of the wild type and triple mutant.

**Additional file 6: Fig. S6.** A proposed model for the functions and crosstalk of three MAPK pathways. a. Roles of *F. graminearum* MAPKs in fungal development, pathogenicity, DON production and abiotic stress responses. b. Roles of *F. graminearum* MAPKs in fungal-bacterial interaction and fungal-fungal interaction.

**Additional file 7: Table S1.** Primers used in this study.

**Additional file 8: Table S2.** Profiles of the differentially expressed genes in the wild type and *Gpmk1 mgv1 Fghog1* triple mutant.

## Data Availability

All data generated or analyzed during this study are included in this published article and its supplementary information files.
