# Supplementary figures and images for: Deletion of all three MAP kinase genes results in severe defects in stress responses and pathogenesis in Fusarium graminearum
Source: Stress Biol. 2022 Jan 17;2(1):6. doi: 10.1007/s44154-021-00025-y (PMC10441923; doi:10.1007/s44154-021-00025-y)

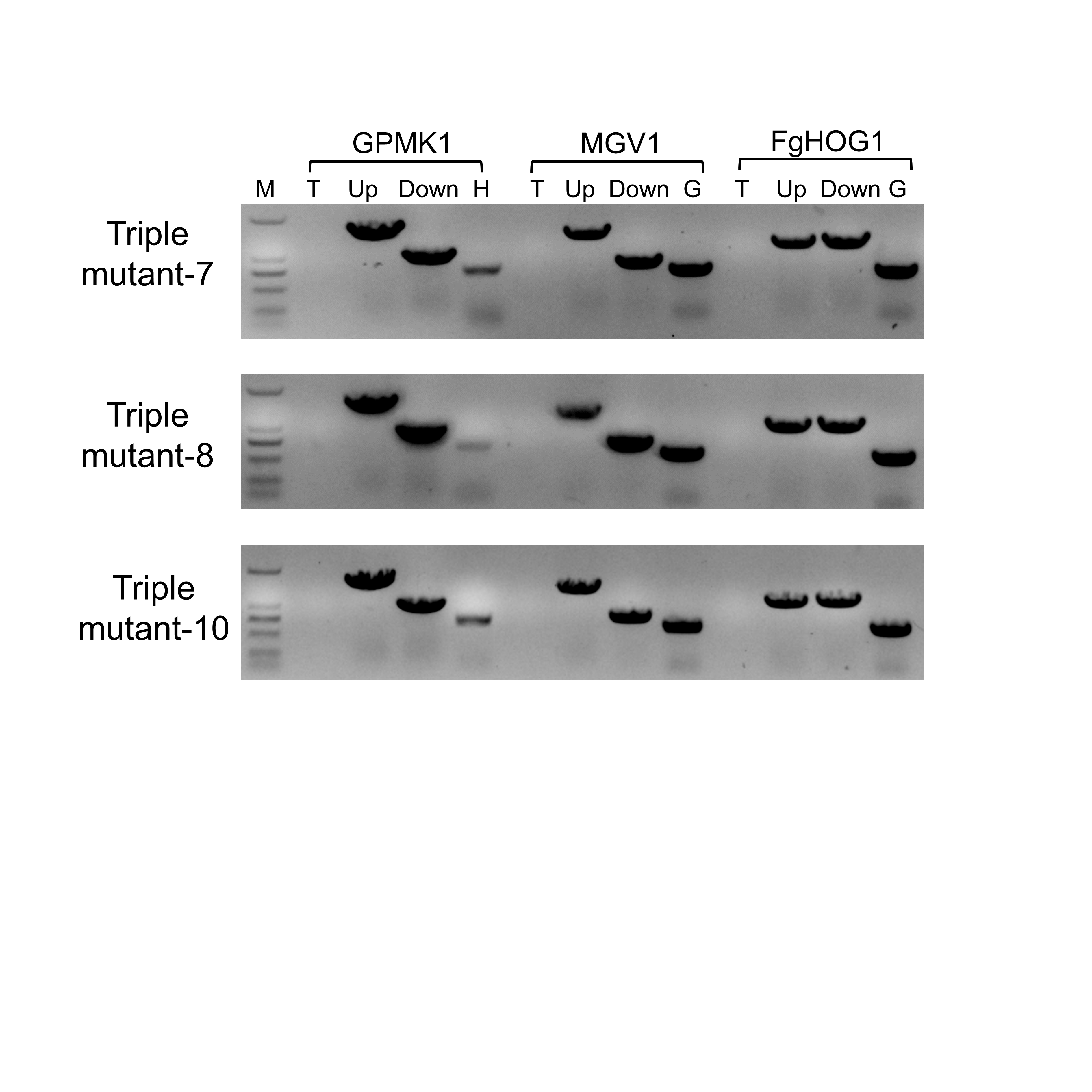

Supplement: Supplementary file 1 — Additional file 1: Fig. S1. Generation of triple deletion mutants of MAPKs. Lane Up and lane Down showed the occurrence of homologous recombination at the upstream and downstream flanking sequences of the labelled MAPK gene, respectively. Lane T shows the deletion of labelled MAPK genes. Lane H or G showed the amplification of selectable marker genes. [file 44154_2021_25_MOESM1_ESM.tif]

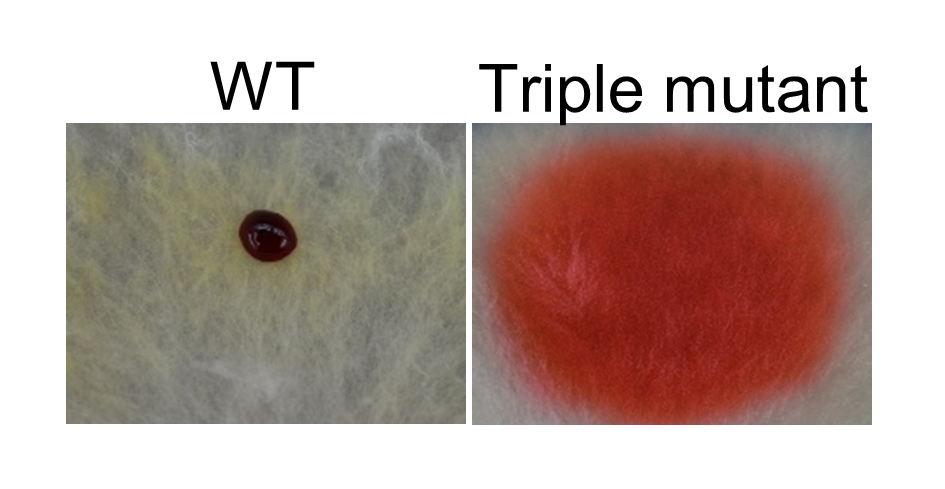

Supplement: Supplementary file 2 — Additional file 2: Fig. S2. Surface hydrophobicity assays with the wild type and triple mutant. Photos were taken 15 min after placing droplets of 20 μl red ink on the colony surface. [file 44154_2021_25_MOESM2_ESM.tif]

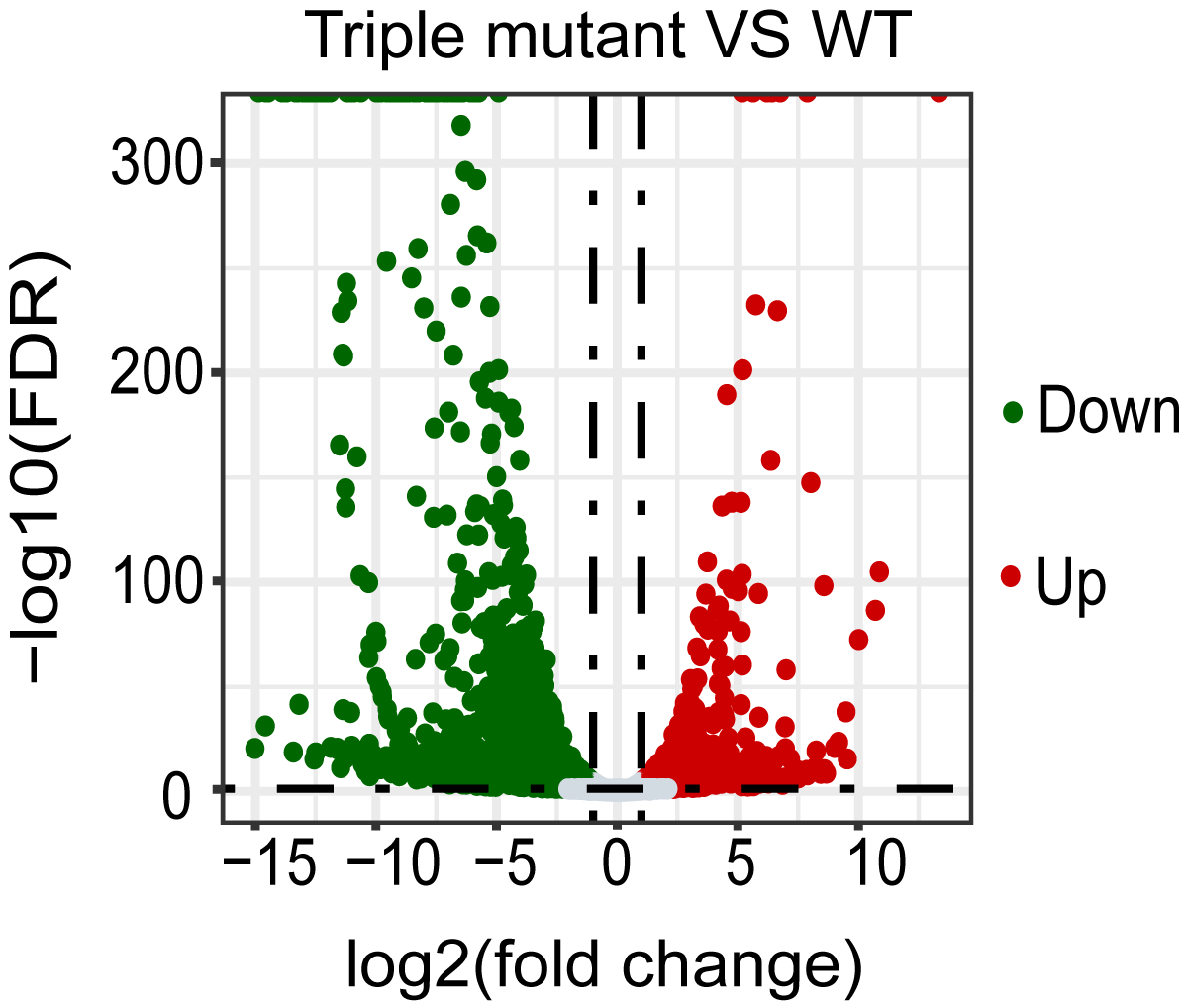

Supplement: Supplementary file 3 — Additional file 3: Fig. S3. A volcano plot of the 1469 up-regulated (red dots) and 2203 down-regulated (green dots) DEGs in Gpmk1 mgv1 Fghog1 triple mutant compared to the wild type. [file 44154_2021_25_MOESM3_ESM.tif]

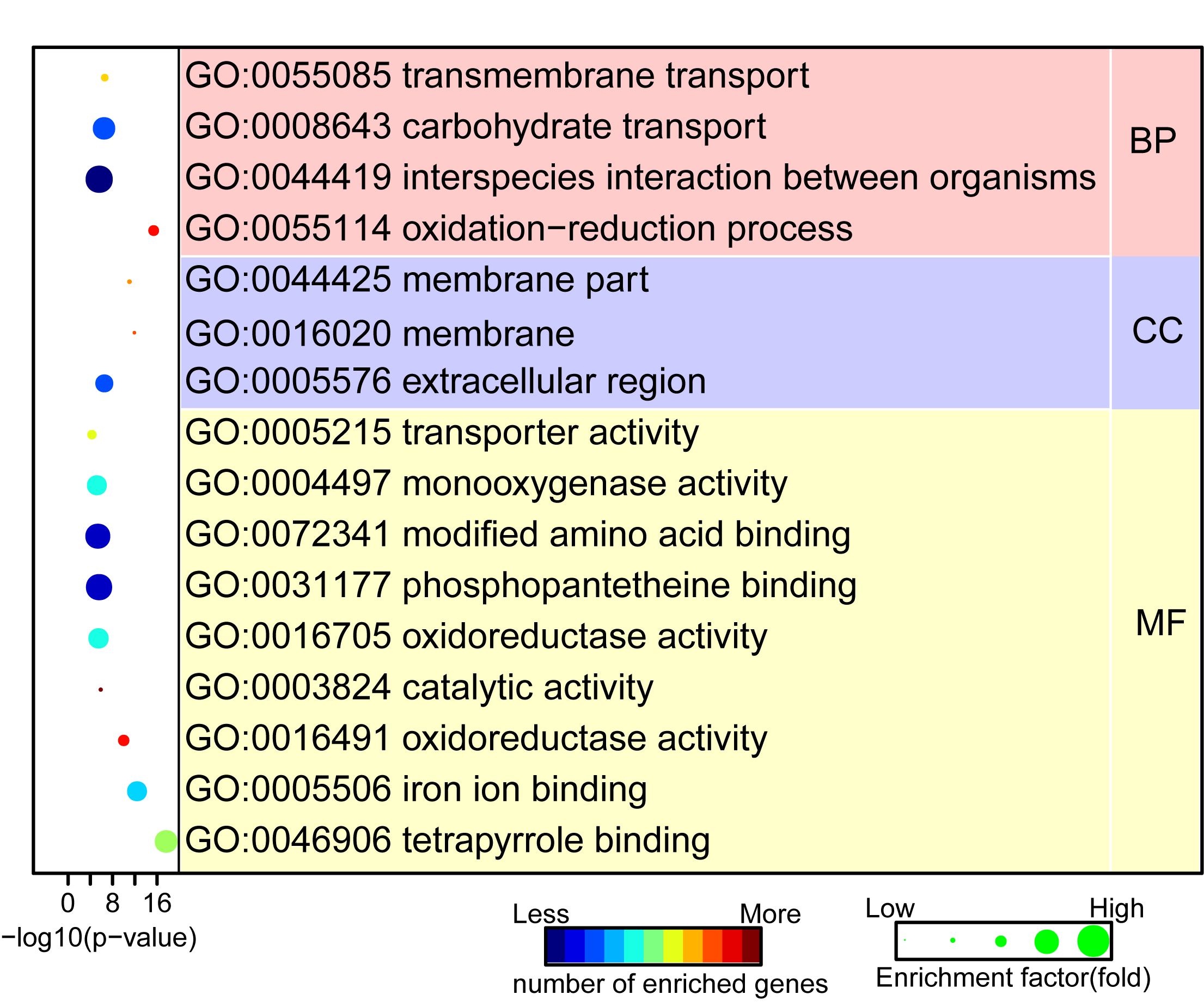

Supplement: Supplementary file 4 — Additional file 4: Fig. S4. GO enrichment analysis of the down-regulated DEGS in the triple mutant. BP, Biological Process; CC, Cellular Components; MF, Molecular Function. [file 44154_2021_25_MOESM4_ESM.tif]

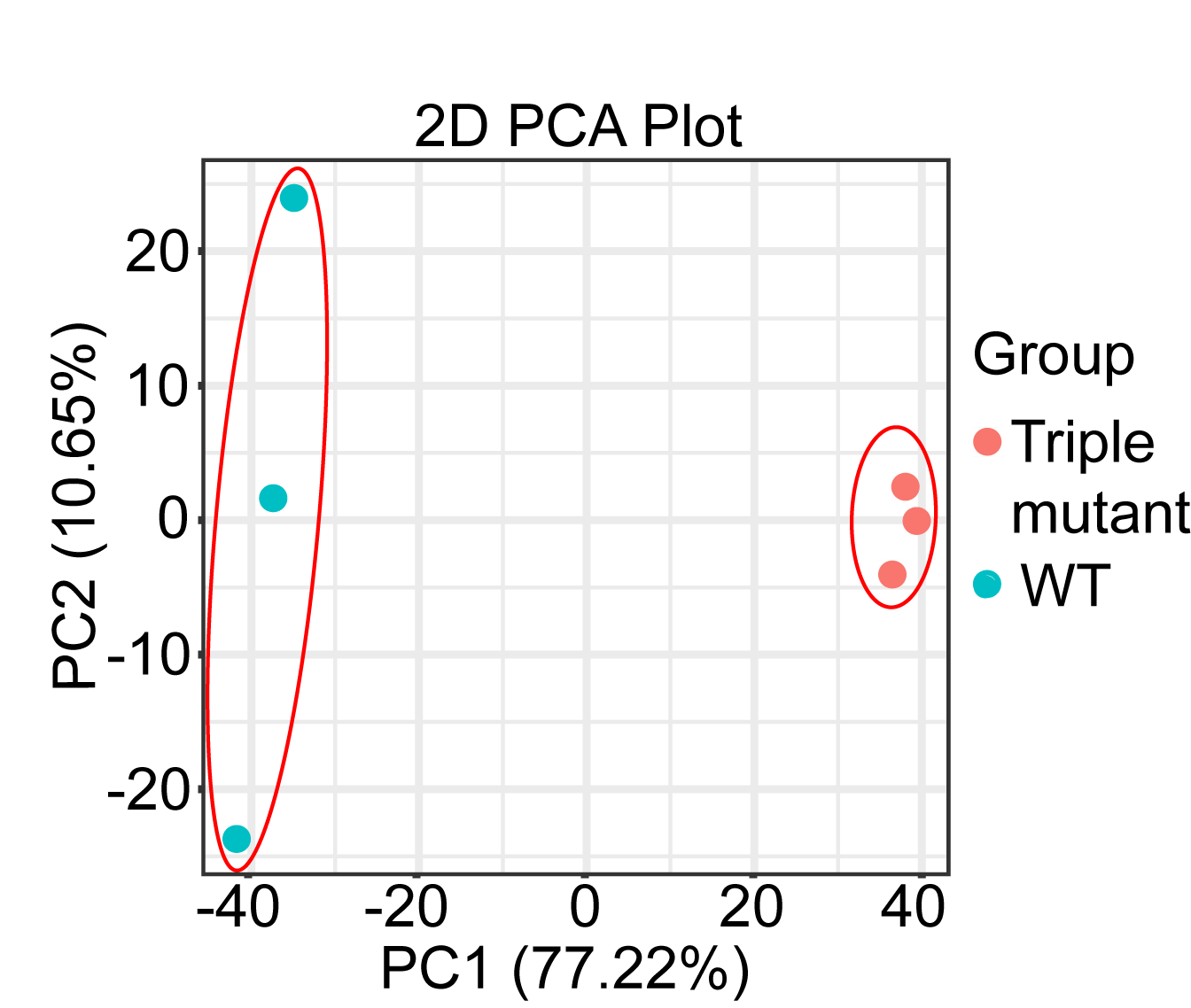

Supplement: Supplementary file 5 — Additional file 5: Fig. S5. Multidimensional scaling plot of the metabolomic profiles of the wild type and triple mutant. [file 44154_2021_25_MOESM5_ESM.tif]

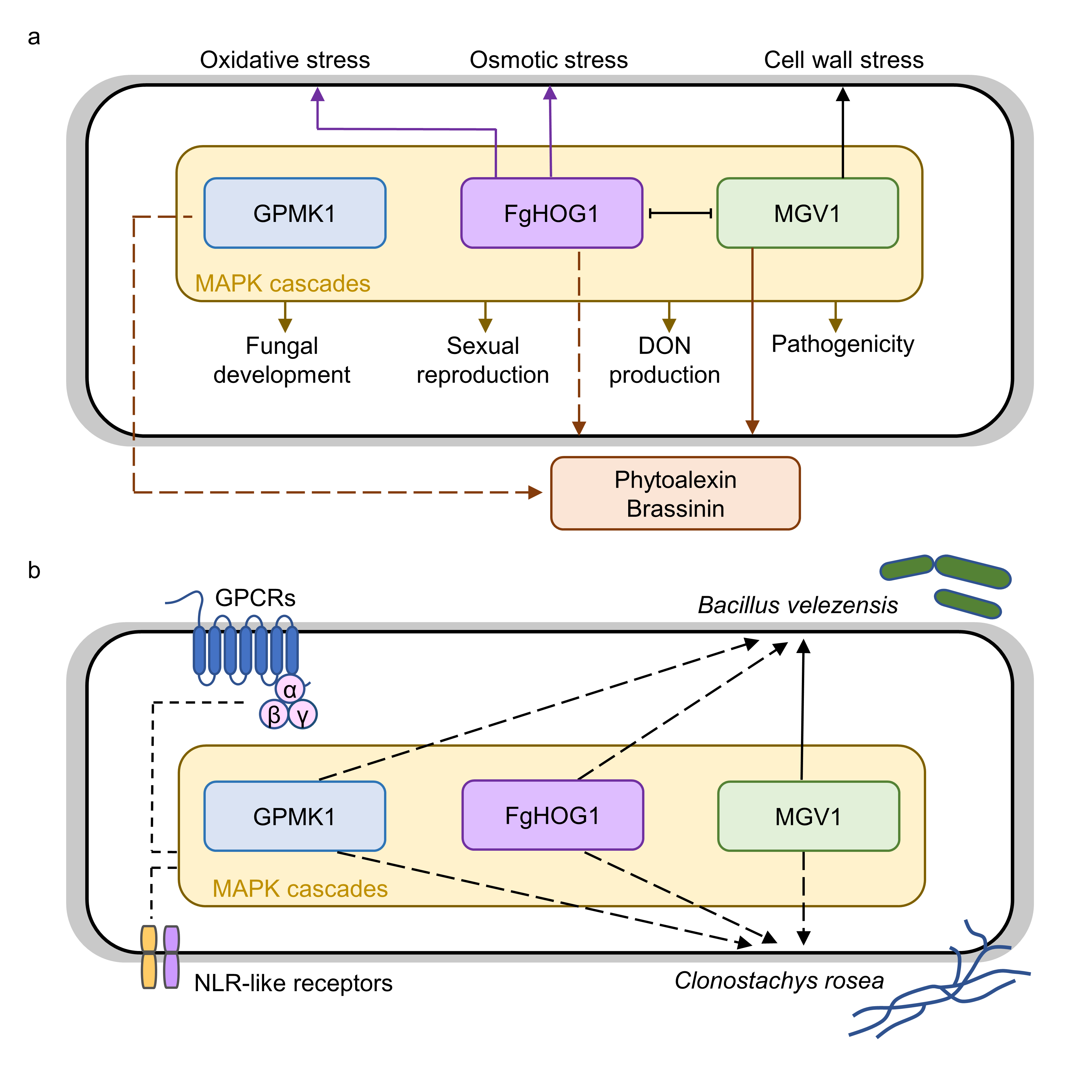

Supplement: Supplementary file 6 — Additional file 6: Fig. S6. A proposed model for the functions and crosstalk of three MAPK pathways. a. Roles of F. graminearum MAPKs in fungal development, pathogenicity, DON production and abiotic stress responses. b. Roles of F. graminearum MAPKs in fungal-bacterial interaction and fungal-fungal interaction. [file 44154_2021_25_MOESM6_ESM.tif]
